# Mulberry Fruit Extract Protects against Memory Impairment and Hippocampal Damage in Animal Model of Vascular Dementia

**DOI:** 10.1155/2012/263520

**Published:** 2012-08-16

**Authors:** Pratchaya Kaewkaen, Terdthai Tong-un, Jintanaporn Wattanathorn, Supaporn Muchimapura, Wiroje Kaewrueng, Sathaporn Wongcharoenwanakit

**Affiliations:** ^1^Department of Physiology and Graduate School (Neuroscience Program), Faculty of Medicine, Khon Kaen University, Khon Kaen 40002, Thailand; ^2^Integrative Complementary Alternative Medicine Research Group, Khon Kaen University, Khon Kaen 40002, Thailand; ^3^Department of Physiology, Faculty of Medicine, Khon Kaen University, Khon Kaen 40002, Thailand; ^4^The Queen Sirikit Department of Sericulture, Ministry of Agriculture and Cooperatives, Bangkok 10900, Thailand

## Abstract

Nowadays, the preventive strategy of vascular dementia, one of the challenge problems of elderly, has received attention due to the limitation of therapeutic efficacy. In this study, we aimed to determine the protective effect and possible mechanism of action of mulberry fruit extract on memory impairment and brain damage in animal model of vascular dementia. Male Wistar rats, weighing 300–350 g, were orally given mulberry extract at doses of 2, 10 and 50 mg/kg at a period of 7 days before and 21 days after the occlusion of right middle cerebral artery (Rt.MCAO). It was found that rats subjected to mulberry fruits plus Rt.MCAO showed the enhanced memory, the increased densities of neuron, cholinergic neuron, Bcl-2-immunopositive neuron together with the decreased oxidative stress in hippocampus. Taken all data together, the cognitive enhancing effect of mulberry fruit extract observed in this study might be partly associated with the increased cholinergic function and its neuroprotective effect in turn occurs partly via the decreased oxidative stress and apoptosis. Therefore, mulberry fruit is the potential natural cognitive enhancer and neuroprotectant. However, further researches are essential to elucidate the possible active ingredient.

## 1. Introduction

Vascular dementia has been recognized as the second most common dementia in elderly. Its prevalence is increased in accompany with the dramatic increase in elderly population. It has been reported that the prevalence of vascular dementia in the elderly is approximate 1.2 to 4.2% and it accounts for 10 to 50% of dementia cases [1]. It occurs as a result of ischemic injury or oligaemia to brain areas involved in cognition, memory, and behavior leading to a progressive cognition decline, functional ability impairment, and behavioral problems [2]. Recent findings point out that brain damage induced in this condition is associated with oxidative stress [3], cholinergic dysfunction [4, 5], and apoptosis [6]. Since, there is no cure for vascular dementia available until now, the focus is currently on preventing further brain damage.

According to Traditional Chinese Medicine (TCM), vascular dementia is considered as a result of insufficiency of kidney-yin and yin-blood [7]. It is believed that yin tonification or a therapeutic treatment that nourishes and replenishes yin of the body when it is deficient or weak can improve yin deficiency condition. Yin tonification can be induced by various methods. However, dietary therapy is a method that is very much easy to approach. According to TCM, food is categorized as “Yin” or “Yang” food according to their characteristics. Since, “Yin foods” such as food with blue or black colors can be used to treat people with yin deficiency, the benefit of consumption of “Yin foods” to prevent vascular dementia has been considered. 

*Morus alba* Linn. or Mulberry, an economic plant in the Northeast of Thailand, is belonging to the family of Moraceae. It is classified in the modern Chinese Materia Medica as a blood tonic. Therefore, it can nourish and promote production of body fluid.

Traditionally, mulberry fruit has been used as a medicinal agent to nourish the yin and blood, benefit the kidneys, and treat weakness, fatigue, anemia, and premature graying of hair. It is also utilized to treat urinary incontinence, tinnitus, dizziness, and constipation in the elderly and the anemic. Recently, it was found that anthocyanin, a flavonoid pigments in mulberry fruit, could protect against cerebral ischemia [8]. Mulberry fruit also possesses anti-inflammatory [9]. In addition, it also possesses antioxidant activity and exert protective against oxidative stress related disease such as Parkinson's disease [10]. Based on Yin property and antioxidant activity of mulberry, we hypothesized that mulberry fruit might prevent against vascular dementia induced by cerebral ischemia.

## 2. Materials and Methods

### 2.1. Animals

Adult male Wistar rats (300–350 g, 8 weeks old) were obtained from National Laboratory Animal Center, Salaya, Nakorn Pathom and were housed in group of 5 per cage in standard metal cages at 22 ± 2°C on 12 : 12 h light-dark cycle. All animals were given access to food and water *ad libitum*. The experiments were performed to minimize animal suffering in accordance with the internationally accepted principles for laboratory use and care of European Community (EEC directive of 1986; 86/609/EEC). The experimental protocols were approved by the Institutional Animal Care and Use Committee (AEKKU 1/2552).

### 2.2. Plant Preparation

All mulberry fruits used in this study are prepared and provided by the Queen Sirikit Department of Seri Culture, Thailand. Mulberry fruits were collected from the Queen Sirikit Seri Culture Center Udon Thani. All berries were picked at the commercially ripen stage and selected according to uniformity color. Then, the fruits were dried at 70°C for 4 days and grounded to powder. Then, 4 kilograms of mulberry fruit powder were extracted 3 times with ethyl alcohol 5 liters per time by percolation techniques. The obtained extracts were evaporated under reduced pressure to yield 7.37% of ethanol extract.

### 2.3. The Experimental Design

Rats were randomly divided into various groups as described following. (1) Vehicle + MCAO; (2) donepezil + MCAO (positive control); (3) Mulberry fruits (2 mg/kg BW) + MCAO; (4) Mulberry fruits (10 mg/kg BW) + MCAO; (5) Mulberry fruits (50 mg/kg BW) + MCAO. All animals were treated with vehicle or positive control or mulberry fruits extract at a period of 1 week before and 3 weeks after right middle cerebral artery occlusion (MCAO).

### 2.4. Surgical Procedure to Induce Vascular Dementia

Focal cerebral ischemia was performed according to modified method of Longa [11]. In brief, rats were anesthetized by thiopental sodium at dose of 50 mg/kg BW. The right common carotid artery and the right external carotid artery were exposed through a ventral midline neck incision and were ligated proximally. A silicone coated nylon monofilament (4–0) suture (USS DGTM sutures; Tyco Healthcare group LP, Connecticut, USA) with its tip rounded by heating near a flame was inserted through an arteriectomy in the common carotid artery just below the carotid bifurcation and then advanced into the internal carotid artery approximately 17 mm distal to the carotid bifurcation until a mild resistance was felt. Occlusion of the origins of the anterior cerebral artery, the middle cerebral artery, and the posterior communicating artery was thereby achieved. Then, the wound was sutured, the rats were returned to their cages with free access to food and water. The incision sites were infiltrated with 10% povidone-iodine solution for antiseptic postoperative care.

### 2.5. Determination of Total Phenolics Compound

The total phenolics compound concentration was measured by a modified Follin-Ciocalteu colorimetric method [12]. Briefly, a sample diluted was added to a test tube containing 1.58 mL of distilled water. Folin-Ciocalteu reagent of 100 *μ*L was added, and the tube was stirred and allowed to stand at room temperature for 8 min. 300 *μ*L of Na_2_CO_3_ (7%, w/v) was added to the mixture and the absorbance was measured at 765 nm after 120 min at room temperature using a spectrophotometer. The results were expressed as milligram of gallic acid equivalents (GAE) per 100 gram fruit (mg GAE/100 g fruit).

### 2.6. Determination of Total Flavonoid Content

Total flavonoid content was determined by using a colorimetric assay [13]. The absorbance of the solution was measured versus a blank at 433 nm using a spectrophotometer. The results were expressed as mg of quercetin equivalents (QE) per 100 gram fruit (mg QE/100 g fruit).

### 2.7. Determination of Total Anthocyanins

Total anthocyanins were estimated by a pH-differential method [14]. Two dilutions of plants were prepared, one with potassium chloride buffer (pH 1.0) (0.189 g KCl in 100 mL of distilled water, pH value adjusted to 1.0 with concentrated HCl), and the other with sodium acetate buffer (pH 4.5) (5.669 g CH_3_CO_2_Na·3H_2_O in 100 mL of distilled water, pH value adjusted to 4.5 with concentrated HCl), absorbance was measured simultaneously at 520 and 700 nm after 15 min incubation at room temperature. The content of total anthocyanins was expressed in mg of cyanidin-3-glucoside equivalents (CGE) per kg of berries using a molar extinction coefficient (*ε*) of cyanidin-3-glucoside of 26900 L mol^−1^ cm^−1^ and molar weight (MW) (449.2 g mol^−1^). Data presented are mean ± standard error of mean (SEM).

### 2.8. Determination of Antioxidant Activity by the 2,2-Diphenyl-1-Picrylhydrazyl (DPPH) 

DPPH assay was determined by previously method described [14]. Each sample (0.5 mL) was added to 0.5 mL of 0.4 mM DPPH in methanol. The mixture was shaken vigorously and allowed to stand for 30 min; the absorbance of the resulting solution was measured at 517 nm with a spectrophotometer. Percent inhibition of DPPH radical was calculated for each dilution of berry extract according to formula: % inhibition = [(A_DPPH_−A_plant_) /A_DPPH_)] × 100, where A_DPPH_ is the absorbance value of the DPPH versus blank solution and A_plant_ is absorbance value of the sample solution. A lower level of absorbance indicated a stronger radical scavenging activity.

### 2.9. Determination of Antioxidant Activity by Ferric Reducing Antioxidant Power (FRAP)

Ferric reducing antioxidant power assay was carried out as previously described [15] with some modifications. The stock solutions were included 300 mM acetate buffer (3.1 g C_2_H_3_NaO_2_·3H_2_O and 16 mL C_2_H_4_O_2_), pH 3.6, 10 mM tripyridyltriazine (TPTZ) solution in 40 mM HCl, and 20 mM FeCl^3^·6H_2_O solution. The fresh working solution was prepared by mixing 25 mL acetate buffer, 2.5 mL TPTZ solution, and 2.5 mL FeCl^3^·6H_2_O solution. The plant extract (10 *μ*L) in 1 mL distilled water was allowed to react with 1.8 mL of the FRAP solution for 10 min at 37°C. The absorbance of the tested solution was monitored at 593 nm. Results are expressed in *μ*M Ascorbic acid/100 g fresh weight.

### 2.10. Assessment of Cognitive Function

Animals were tested spatial memory by the Morris water maze test [16]. The apparatus was a pool with 170 cm diameter filled up with tap water for 40 cm deep and the water surface was covered with nontoxic powder. The pool was divided into four quadrants and the removable escape platform was placed in the center on one quadrant below the water level. For animals, the location of the platform was invisible and it remained there throughout the training. The animals must memorize the environment cues to locate the platform. Each animal was placed in the water in the starting quadrant and allowed to swim until it found and climbed onto the platform. The time for animal to reach the hidden platform was recorded as escape latency or acquisition time.

### 2.11. Determination of Scavenging Enzymes and the Malondialdehyde Level

After the last dose of administration, all rats were sacrificed. The hippocampus of the lesion side was isolated and prepared as a homogenate to determine superoxide dismutase (SOD) activity was estimated by the method of McCord and Fridovich [17] while catalase and glutathione peroxidase activities were determined by method of Aebi et al. [18] and Dundar et al. [19], respectively. In addition, the MDA and AChE were estimated by determining the accumulation of thiobarbituric acid reactive substances (TBARS). The activities of all enzyme mentioned above were expressed as U/mg protein.

### 2.12. Histological and Immunohistochemical Studies

#### 2.12.1. Cresyl Violet Staining for Nissl Substance

Adjacent series of sections hippocampus from control, sham operate, vehicle, Aricept, vitamin C, *Morus alba *treated group stained with 0.5% cresyl violet to aid in neuronal death density determination. All sections of hippocampus had elevated with the aid Olympus light microscope model BH-2 (made in Japan). To determine the density of neurons, fine representative nonadjacent sections contain hippocampus had been selected for analysis. The observer blinded to the treatment at time of analysis. The density of neuron cell death had determined at 40X magnification.

#### 2.12.2. Immunohistochemical Staining of Bcl-2 Immunopositive Neurons and Cholinergic Neurons Density

A series of sections containing hippocampus were reacted in a mouse monoclonal antibody directed against Bcl-2 (Chemicon Internation, Inc., CA, USA) and a modification of a previously described protocol employing the DAKO Strept ABC Complex/HRP duet kit. In brief, the sections were eliminated endogenous peroxidase activity by 0.5% H_2_O_2_ in methanol. Sections were washed in running tap water and distilled water for 1 minute each, then rinsed in KPBS and KPBS-BT for 5 minutes per each process. Excess buffer was removed, and then incubated for 30 minutes in a blocking solution composed of 5% normal goat serum in KPBS-BT. Then, the sections were incubated in mouse primary antibody against Bcl-2 diluted 1 : 400 in KPBS-BT at room temperature for 2 hours and incubate at 4°C for 48 hours. The tissue was rinsed in KPBS-BT (2 washes × 7 minutes), incubated for 1 hours in biotinylated goat antimouse IgG antibody, rinsed in KPBS-BT (2 washes × 7 minutes), and then incubated in Strept ABC Complex/HRP for 4 hours. The sections were rinsed in KPBS-BT (1 minutes), and KPBS (2 washes × 10 minutes). Bcl-2 immunoreactivity was visualized using 0.025% 3,3′diaminobenzedine (DAB, Sigma) and 0.01% H_2_O_2_. for 24 hours. Finally, sections were rinsed in running tap water, air dried, and cover-slipped using permount.

According to this part, the mouse monochonal antibody direct against choline acetyltransferase (ChAT) were use instead of monochonal antibody direct against Bcl-2.

### 2.13. Statistical Analysis

Data were presented as mean ± standard error of mean (SEM). The analysis was performed using one-way analysis of variance (ANOVA), followed by LSD test. All statistical results were considered significant at *P* value < .05

## 3. Results

### 3.1. The Contents of Phenolic Compounds, Total Flavonoids, Anthocyanin, and Antioxidant Effect of Mulberry Fruit

In the first part of this study we had determined and compared the phenolic compounds, total flavonoids, and anthocyanin contents of mulberry fruits with the fruits which reputed for anthocyanin riches and possesses neuroprotective and cognitive enhancing effects as blueberries. In addition, the antioxidant effect was also determined using DPPH and FRAP assays. The results were shown in Table 1. Our data clearly demonstrated that dried powder of mulberry fruits contained phenolic compounds at concentration of 5.19 mg GAE/g of fruit whereas blueberry fruits contained the mentioned compounds at concentration of 2.25 mg GAE/g of fruit. The contents of total flavonoids in the forms of quercetin of mulberry fruits and blueberry fruits were observed at concentrations of 9.44 and 2.74 mg QE/100 g of fruit while total flavonoids which determined in the form of rutin contents were observed at concentrations of 6.92 and 1.45 mg Rutin/100 g of fruit. Therefore, mulberry fruits contained more phenolic compounds than blueberry fruits approximately 43%. Mulberry fruits also contained more quercetin and rutin than blueberry fruits around 29 and 20%. We also determined the contents of anthocyanin in both types of fruit. It was found that anthocyanin contents in mulberry and blueberry fruits were 667.95 and 333.98 mg/100 g of fruit. Again, mulberry fruits showed higher concentration of anthocyanin more than blueberry fruits approximately 50%. Therefore, it is very interesting that mulberry fruit which is very much cheaper than blueberry fruit contains more benefit phytochemical ingredients. However, health benefit of mulberry fruit has not received much attention.

### 3.2. Cognitive Enhancing Effect and Neuroprotective Effect of Mulberry Fruit

**Figure 1:**
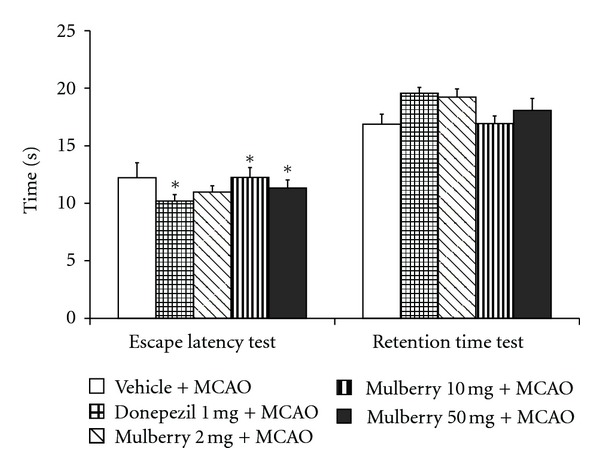
The effect of mulberry fruit extract on spatial memory in animal model of vascular dementia.

The effect of mulberry fruit extract on spatial memory in animal model of vascular dementia was shown in Figure 1. Our data showed that at 21-day after MCAO, rats subjected to donepezil, a standard drug for dementia treatment which was used as positive control showed the significant decreased escape latency (*P* value < .01 compared to MCAO + vehicle treated group) whereas no significant change of retention time was observed. Rats which obtained mulberry fruit extract at doses of 10 to 50 mg/kg BW also showed the significant decreased escape latency (*P* value < .05 all; compared to MCAO + vehicle treated group). However, no significant changes of retention time were observed in any groups.

**Figure 2:**
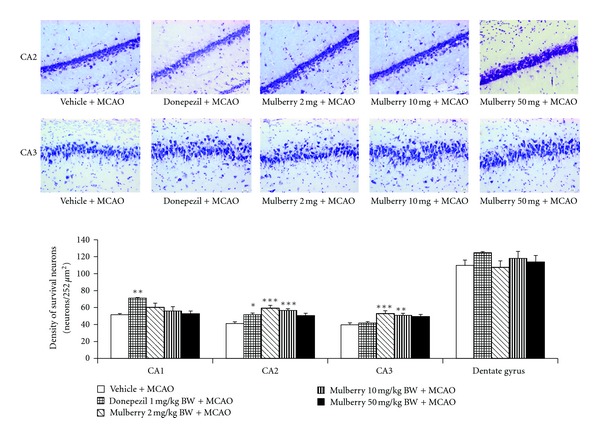
The effect of mulberry fruit extract on neuron density in hippocampus (Photomicrographs of coronal sections of rat brain histologically stained with cresyl violet at 40X magnification).

Figure 2 showed the effect of mulberry fruit extract on neuron density in hippocampus. The results showed that rats which received donepezil showed the significant elevation of neuron density only in CA1 (*P* value < .01 compared to MCAO + vehicle treated group). Surprisingly, rats subjected to all doses of mulberry fruit extract significantly enhanced neuron density in CA2 (*P* value < .001, .001 and .05 respectively; compared to MCAO + vehicle treated group) and CA3 (*P* value < .001, .01, and .01, respectively; compared to MCAO + vehicle treated group) whereas no significant changes were observed in CA1 and dentate gyrus.

**Figure 3:**
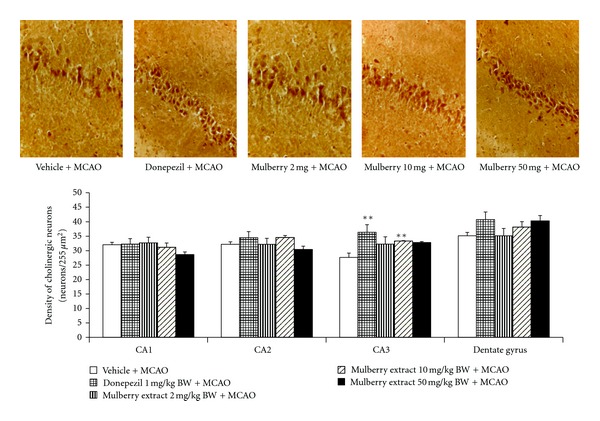
The effect of mulberry fruit extract on alteration of density of cholinergic neuron in various subregion of hippocampus (Photomicrographs of coronal sections of rat brain (panel A = CA3 of hippocampus) immunohistologically stained with cholineacetyl transferase at 40X magnification).

**Figure 4:**
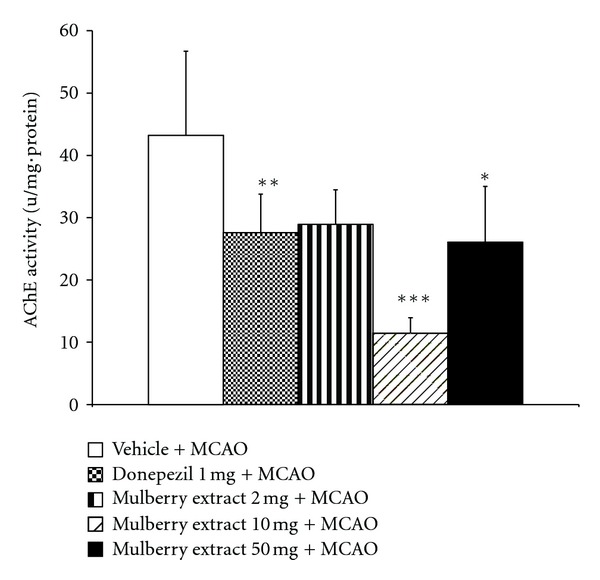
The effect of mulberry fruit extract on the alteration of acetylcholinesterase enzyme activity.

We also determined the effect of mulberry fruits extract on cholinergic system. It was found that rats which received either donepezil or mulberry fruit extract at dose of 10 mg/kg BW showed the increased neuron density in CA3 subregion of hippocampus (*P* value < .01 all compared to MCAO + vehicle treated group) as shown in Figure 3. In addition, the effect of mulberry fruit on the activity of AChE in hippocampus was also investigated and data were shown in Figure 4. The present findings showed that rats which received either donepezil or mulberry fruit at doses of 10 and 50 mg/kg BW showed the significant reduction of AChE activity in hippocampus (*P* value < .01 and .05, respectively; compared to MCAO + vehicle treated group).

### 3.3. Effect of Mulberry Fruit on Bcl-2-Immunopositive Neurons Density

**Figure 5:**
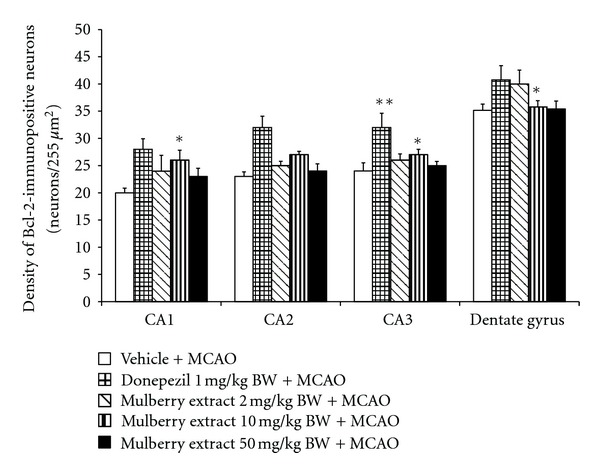
The effect of mulberry fruit on Bcl-2-immunopositive neurons density in various subregion of hippocampus.

Since apoptosis also contributed the crucial role on brain damage in vascular dementia, we also focused on the alteration of Bcl 2, the apoptosis regulator. The effect of mulberry fruit extract on the density of Bcl-2-immunopositive neurons in hippocampus was investigated and data were shown in Figure 5. Rats which exposed to donepezil showed the increased Bcl-2-immunopositive neurons density only in CA3 (*P* value < .05 compared to MCAO + vehicle treated group). It was found that rats subjected to mulberry fruit extract at dose of 10 mg/kg BW showed the significant increase in Bcl-2-immunopositive neurons density in CA1 and CA3 (*P* value < .05 all; compared to MCAO + vehicle treated group) while rats which received the extract at dose of 2 mg/kg BW showed the significant increase Bcl-2-immunopositive neurons density only in dentate gyrus (*P* value < .05 compared to MCAO + vehicle treated group).

### 3.4. Effect of Mulberry Fruit Extract on Oxidative Stress Markers

In this study, we also determined the effect of mulberry fruits extract on oxidative stress markers including the level of MDA and the activities of superoxide dismutase (SOD), catalase (CAT) and glutathione peroxidase (GSH-Px). The results were shown in Table 2. It was found that both rats subjected donepezil and rats subjected to all doses of extract significantly decreased MDA level in hippocampus (*P* value < .001, .05, .01, and .01, respectively; compared to MCAO + vehicle treated group) but increased CAT activity in the mentioned area (*P* value < .05, .001, .001, and .001, respectively; compared to MCAO + vehicle treated group). The enhanced activity of SOD in hippocampus was also observed in rats treated with donepezil and mulberry fruit extract at doses of 2, 10 and 50 mg/kg BW (*P* value < .05 all; compared to MCAO + vehicle treated group). The enhanced GSH-Px activity was also observed in rats treated with mulberry fruit at dose of 10 and 50 mg/kg (*P* value < .05.all; compared to MCAO + vehicle treated group).

## 4. Discussion

The present study has revealed that mulberry fruit possessed higher contents of phytochemical compounds and exerted more potent antioxidant activity than blueberry fruit. We have clearly demonstrated that mulberry fruit extract significantly improved oxidative status and enhanced the densities of neuron and cholinergic neuron in hippocampus. In addition, the enhanced density of Bcl-2-immunopositive neurons and the suppression of AChE in the area just mentioned were also observed in accompany with the increased spatial memory in animal model of vascular dementia.

The present study has revealed that mulberry fruit possessed higher contents of phytochemical compounds and exerted more potent antioxidant activity than blueberry fruit. We have clearly demonstrated that mulberry fruit extract significantly improved oxidative status and enhanced the densities of neuron and cholinergic neuron in hippocampus. In addition, the enhanced density of Bcl-2-immunopositive neurons and the suppression of AChE in the area just mentioned were also observed in accompany with the increased spatial memory in animal model of vascular dementia.

In our behavioral test, we found that mulberry fruit extract could improve memory impairment in animal model of vascular dementia. To further elucidate the possible mechanism associated with the recovery of memory impairment in cerebral ischemia rat, we had determined the function of cholinergic system which played the pivotal role on learning and memory in the hippocampus. We had found that the function of cholinergic system in this area increased by the enhanced cholinergic neuron and the suppression of AChE. Since cholinergic dysfunction played an important role in the pathophysiology of vascular dementia [20], therefore, the improved memory impairment induced by mulberry fruit might occur partly via the enhanced cholinergic function.

In addition the enhanced neuron density in hippocampus was also observed especially in CA2 and CA3. It has been reported that CA3 and CA1 contribute the important role in associative memory function. Both areas also play the role in encoding process while only CA3 also play a pivotal role in retrieval process [21, 22]. Unfortunately, the function of CA2 has not been yet understood. The increased neuron density observed in this study might be associated with the decreased oxidative stress induced by the elevation of SOD, GSH-Px, and CAT in hippocampus or the ability of mulberry fruit to directly scavenge the stable free radicals and its reducing power. However, the role of mulberry fruits on apoptosis still could not omitted. 

Previous study had demonstrated that memory impairment in MCAO rat was associated with neuronal apoptosis in hippocampus [23]. In addition, it was also reported that this process can be suppressed by the enhanced expression of Bcl-2. [24, 25]. In this study, we found that Bcl-2 expression was significantly increased in rats subjected to mulberry fruits extract + MCAO rats. Therefore, this finding suggested that the neuroprotective effect of mulberry fruit against apoptosis in rat hippocampal was also mediated partly by the increased Bcl-2-immunopositive neurons density.

Taken all together, mulberry fruit might decrease oxidative stress and increase Bcl-2 resulting in the increased densities of neurons and cholinergic neurons in hippocampus especially in CA3. Both the increased neuron density in CA3 and the increased cholinergic function gave rise to the increased encoding and retrieval capacity and finally resulted in the enhanced memory in animal model of vascular dementia.

Although the determination of possible active ingredient was beyond the scope of this study, we did suggest that the beneficial effect of mulberry fruit might be associated with its phytochemical compounds such as polyphenolic compounds especially anthocyanin, quercetin, and rutin [26–29].

## 5. Conclusions

Mulberry fruit is the potential functional food that can protect against brain damage and memory impairment in vascular dementia. The possible underlying mechanisms are associated with the improved cholinergic function and the decreased apoptosis by enhancing the Bcl-2 immunospositive neuron density in hippocampus. In addition, the decreased oxidative stress by the enhanced SOD and CAT activities in hippocampus and the direct ability to scavenge stable oxidative stress and reducing power of mulberry fruits may also contribute the role. The decreased oxidative stress and apoptosis give rise to the enhanced neuron density in hippocampus especially CA3 which in turn increase the encoding and retrieval capability of learning and memory process and finally lead to the improved memory impairment. However, further researches about possible active ingredient is still required.

## Figures and Tables

**Table 1:** The phenolic compounds, total flavonoids, and anthocyanin contents of mulberry and blueberries.

Plants	Total phenolics	Total flavonoids	Total flavonoids	Total anthocyanins	DPPH EC_50_	FRAP EC_50_
	(mg GAE/100 g fruit)	(mg QE/100 g fruit)	(mg Rutin/100 g fruit)	(mg/100 g fruit)	(*μ*g/mL)	(*μ*g/mL)
Mulberry	5.19	9.44	6.92	667.95	232.25	422.28
Blueberry	2.25	2.74	1.45	333.98	574.38	>1000.00

**Table 2:** Effect of mulberry fruit extract on oxidative stress markers.

Group	SOD	Catalase	GSH-Px	MDA
	(*μ*/mg·protein)	(*μ*/mg·protein)	(*μ*/mg·protein)	(*μ*/mg·protein)
Vehicle + MCAO	1.65 ± 0.18	12.84 ± 1.54	0.52 ± 0.10	2.45 ± 0.80
Donepezil 1 mg + MCAO	1.82 ± 0.33	36.46 ± 7.11***	0.95 ± 0.28	1.68 ± 0.39
Mulberry 2 mg + MCAO	2.65 ± 0.41	15.22 ± 4.05	0.78 ± 0.22	1.55 ± 0.32
Mulberry 10 mg + MCAO	2.47 ± 0.55	19.80 ± 6.65	1.19 ± 0.36*	0.98 ± 0.28*
Mulberry 50 mg + MCAO	2.77 ± 0.51*	13.88 ± 3.64	1.33 ± 0.16*	1.51 ± 0.25

Data are the mean ± SEM. *P < .05, **P < .01, ***P < .001 compared with vehicle treated group.

Abbreviations: SOD: Superoxide dismutase; GSH-Px: Glutathione peroxidase; MDA: Malondialdehyde.
